# Sex Differences in Older Adults with Risky Alcohol Consumption: Medication Use, Comorbidities, and Hepatic Biomarkers

**DOI:** 10.3390/nu18142346

**Published:** 2026-07-17

**Authors:** Ingrid Arteaga, Meritxell Carmona-Cervelló, Guillem Pera, Carla Chacón, Galadriel Diez-Fadrique, Irene Ruiz-Rojano, Maria Palau-Antoja, Faranak Nooriankafshgari, Cristina Vedia, Pilar Montero-Alía, Maria Carmen Rodríguez-Pérez, Susana Montesinos, Pere Torán-Monserrat

**Affiliations:** 1 Unitat de Suport a la Recerca Metropolitana Nord (USR MN), Fundació Institut Universitari d’Investigació en Atenció Primària Jordi Gol i Gurina (IDIAP Jordi Gol), 08303 Mataró, Spain; 2 Multidisciplinary and Translational Research Group on Health and Society in Primary Health Care (GREMSAS-TranslAP), Fundació Institut Universitari d’Investigació en Atenció Primària Jordi Gol i Gurina (IDIAP Jordi Gol), USR MN, 08303 Mataró, Spain; 3 Primary Healthcare Center Vall del Tenes, Gerència d’Àmbit d’Atenció Primària Metropolitana Nord, Institut Català de la Salut (ICS), 08186 Lliçà d’Amunt, Spain; 4 Germans Trias i Pujol Research Institute (IGTP), 08916 Badalona, Spain; 5 Primary Healthcare Center Doctor Guillermo Masriera i Guardiola, Gerència d’Àmbit d’Atenció Primària i a la Comunitat Barcelonès Nord i Maresme, Institut Català de la Salut (ICS), 08340 Vilassar de Mar, Spain; 6 Department of Pharmacy, Gerència d’Àmbit d’Atenció Primària i a la Comunitat Barcelonès Nord i Maresme, Institut Català de la Salut (ICS), 08911 Badalona, Spain; 7 Primary Healthcare Center la Riera, Gerència d’Àmbit d’Atenció Primària i a la Comunitat Barcelonès Nord i Maresme, Institut Català de la Salut (ICS), 08302 Mataró, Spain; 8 Primary Healthcare Center Martorelles-Sant Fost, Gerència d’Àmbit d’Atenció Primària i a la Comunitat Vallès Occidental i Vallès Oriental, Institut Català de la Salut (ICS), 08100 Mollet del Vallès, Spain; 9 Department of Medical Sciences, Faculty of Medicine, Universitat de Girona, 17004 Girona, Spain

**Keywords:** alcohol drinking, aged, prescription drugs, sex differences, primary health care

## Abstract

**Background/Objectives**: Alcohol consumption and medication use have increased among older population, potentially increasing the risk of alcohol–drug interactions and adverse outcomes. Biological sex differences may further influence these effects. This study aimed to assess sex-related differences in medication use, comorbidities, and hepatic biomarkers among older adults with risky alcohol consumption. **Methods**: A cross-sectional, multicenter study was conducted in 455 adults aged ≥65 years. Sociodemographic and clinical characteristics, medication prescriptions, and hepatic biomarkers were collected. **Results**: Of the participants, 41% were women, with a mean age of 71 years. The median weekly alcohol consumption was 7 standard drink units. Overall, 90% were taking at least one medication, with antihypertensives being the most prescribed (62%). Significant sex differences were observed (*p* ≤ 0.01): men showed higher use of antidiabetic drugs (24%), anticoagulants (8%), and nitrates (4%), whereas women more frequently used anxiolytics (35%), non-steroidal anti-inflammatory drugs (22%), and antidepressants (18%). Although risky alcohol consumption was more prevalent among men, women had a higher overall medication use. Significant differences were also observed in hepatic biomarkers, with higher gamma-glutamyl transferase levels and a higher prevalence of elevated FIB-4 values among men (*p* ≤ 0.01). **Conclusions**: These findings highlight the importance of routinely assessing alcohol consumption and medication use in older adults and support incorporating a sex-specific perspective into clinical practice and future research.

## 1. Introduction

Alcohol consumption among the elderly has increased in recent decades [1,2,3]. It is estimated that around 8% to 20% of adults aged 65 years and over consume alcohol in excess [4,5,6]. In Spain, one in four older adults consumes alcohol in quantities that exceed the recommended amount [7,8,9]. 

Although the health risks associated with alcohol generally increase with the amount consumed, current international recommendations define low-risk drinking using standard drink units (SDU) and daily or weekly consumption thresholds [10]. However, international thresholds for low-risk alcohol consumption in older adults remain poorly standardized, and there is currently no international consensus regarding how risky alcohol consumption should be defined in this population [11]. Furthermore, accumulating evidence indicates that older adults with chronic diseases or those receiving medications with the potential for alcohol interactions represent a particularly vulnerable population, in whom alcohol consumption may increase the risk of adverse health outcomes [12,13]. Consequently, for older adults with alcohol-sensitive medical conditions or those receiving medications known to interact with alcohol, abstinence may represent the safest clinical recommendation.

Concurrently, medication use has increased, and polypharmacy has become increasingly prevalent among older adults [14]. In the elderly population, polypharmacy, defined as the use of five or more drugs [15], affects approximately 22% of adults aged 65 and older [16].

This context suggests that a significant percentage of older adults consume alcohol while receiving pharmacological treatment. A meta-analysis revealed that between 21% and 35% of older adults consume alcohol concurrently with drugs that may have the potential for interaction [17,18]. In this regard, even moderate alcohol consumption has been associated with a 24% increase in the risk of adverse drug reactions [19]. In addition to these factors, physiological changes related to aging contribute to an increased vulnerability to interactions with substances. These changes include alterations in the distribution and metabolism of substances, as well as a heightened sensitivity of the central nervous system [20,21].

Interactions between alcohol and medications have the potential to interfere with the effectiveness of drugs, cause adverse side effects, reduce or exacerbate their effects, and consequently worsen health problems [18]. Evidence suggests that even low levels of alcohol consumption, or any alcohol intake at all, may produce harmful effects when combined with certain medications [18]. In this context, alcohol has been demonstrated to potentiate the sedative effects of antidepressants, barbiturates, benzodiazepines, and opioids, thereby increasing the risk of falls; exacerbate the gastrointestinal and renal adverse effects associated with nonsteroidal anti-inflammatory drugs (NSAIDs) [22]; and augment the risk of hypoglycemia in diabetics undergoing hypoglycemic treatment [23]. However, the existing literature has yet to explore the differences linked to biological sex in a comprehensive manner.

While research has documented variations in alcohol consumption patterns between men and women, studies have also demonstrated that women exhibit a heightened vulnerability to the effects of alcohol [24]. This vulnerability is partly attributable to biological differences [18], including body composition, pharmacokinetic differences in alcohol metabolism, effects on the brain, and the influence of sex hormones [25]. These disparities also influence the pharmacokinetics and pharmacodynamics of medications, modulating their efficacy, safety, and metabolism [26,27]. However, among individuals with risky alcohol consumption, there is limited evidence regarding sex-related differences in medication use patterns and their association with clinical and biochemical characteristics.

In this context, this study aims to evaluate sex differences in older adults with risky alcohol consumption, considering medication use, comorbidity burden, and liver biomarkers. These findings may inform future clinical strategies aimed at improving the identification, prevention, and management of alcohol consumption among older adults receiving pharmacological treatment.

## 2. Materials and Methods

### 2.1. Study Design and Participants

This community-based study utilizes data from a two-phase population-based ALANE cohort study. The cohort included individuals registered at 25 Primary Health Care centers (PHCs) in the Barcelona Nord and Maresme areas (Catalonia, Spain), recruited between 2015 and 2021. The study area covers a population of approximately 700,000 inhabitants, of whom 82,903 were aged ≥65 years. 

Participants were randomly selected from the Primary Care Information System (SIAP), which includes all individuals with national healthcare cards and is equivalent to the population census of Catalonia. This database includes all individuals assigned to PHCs in the study area, regardless of healthcare utilization. For more details regarding the study design, please refer to the previous publication [28].

The study was approved by the Ethics Committee of the Foundation University Institute for Primary Health Care Research Jordi Gol i Gurina (P10/35, 30 June 2010).

The study population included participants of both sexes who were 65 years of age or older and were assigned to the participating PHCs. Individuals with conditions that could impede data collection or follow-up were excluded from the study. Exclusion criteria included disabling diseases, cognitive impairment, residence in long-stay centers, or a diagnosis of alcohol dependence according to the International Classification of Diseases (ICD-10).

Participants were classified as having risky alcohol consumption according to the following ALANE cohort criteria [28]:

- Males: consumption of more than 1 SDU (equivalent to 10 g of pure alcohol) per day; or more than 7 SDU per week; or more than 2 SDU on one drinking occasion [29]; or any consumption if they have a medical condition that worsens with alcohol consumption or are taking medications that interact with alcohol.
- Females: from 1 SDU (equivalent to 10 g of pure alcohol) per day; or 7 SDU per week; or more than 1 SDU on one drinking occasion [29]; or any consumption if they have a condition that worsens with alcohol consumption or are taking medications that interact with alcohol.

The definition of risky alcohol consumption used in the ALANE cohort was specifically developed for older adults. In addition to alcohol consumption thresholds, it incorporates any alcohol intake in individuals with alcohol-sensitive medical conditions or concomitant use of medications known to interact with alcohol (Supplementary Table S1). This definition reflects the greater clinical vulnerability of older adults, for whom alcohol-related risk is determined not only by the amount consumed but also by age-related physiological changes, multimorbidity, and the potential for alcohol–drug interactions, in line with current clinical recommendations [13,30].

### 2.2. Variables

The following variables were collected: sociodemographic variables (age, sex, and educational level), clinical variables (comorbidities, medication intake recorded in computerized medical history or self-reported, and body mass index), substance use (alcohol and tobacco), and laboratory variables (aspartate aminotransferase (AST), alanine aminotransferase (ALT), and gamma-glutamyl transferase (GGT)).

Alcohol consumption was assessed using a structured questionnaire. Alcohol intake was recorded in SDU, where one SDU corresponds to 10 g of pure alcohol. Participants were asked about the quantity and frequency of alcohol consumption during the previous month, distinguishing between weekday, weekend, and occasional drinking. Information on the type of alcoholic beverage consumed was also collected, and total weekly alcohol consumption was calculated.

Two indices were calculated based on analytical data, to assess the presence of liver disease: 

AST/ALT ratio, also known as the De Ritis index, was calculated as the ratio between AST and ALT values. This index has been shown to be useful in the diagnosis and prognosis of various liver diseases, including alcoholic liver disease [31]. An AST/ALT ratio > 1.5 strongly suggests alcohol-induced liver damage, and a ratio > 2.0 is almost certainly indicative of alcohol-induced liver damage [32]. 

The FIB-4 index is a non-invasive serological marker used to stratify the risk of advanced liver fibrosis in patients with suspected chronic liver disease, such as those with excessive alcohol consumption [33]. The FIB-4 was calculated using the following formula [34]:

$$\mathrm{FIB}\text{-}4=[\mathrm{age}\;(\mathrm{years})\times \mathrm{AST}\;(U/L)]/[\mathrm{platelet}\;\mathrm{count}\;(\times 10^{9}/L)\times \sqrt{ALT(U/L)}]$$

In the population over 65 years of age, a value less than 2.0 allows the presence of advanced liver fibrosis to be ruled out [35].

### 2.3. Data Collection

Participants were selected using random sampling from SIAP, and initial contact was made by telephone. 

Eligible participants who agreed to participate were scheduled for an initial visit at their respective PHCs to identify participants with risky alcohol consumption. During the visit, the inclusion and exclusion criteria were verified, the study information sheet was provided, and written informed consent was obtained.

Following enrolment, sociodemographic, clinical data, and information on alcohol consumption were collected. As part of the original ALANE cohort design, a random subgroup of participants with risky alcohol consumption was selected for blood sampling. However, not all selected participants attended the scheduled blood sampling visit, and some laboratory parameters were unavailable for certain individuals. Consequently, biochemical analyses were performed using all available laboratory data, and the number of observations varied across the different analyses.

### 2.4. Statistical Analysis

Distributions of continuous variables were assessed using histograms. Normally distributed continuous variables are presented as means and standard deviations, while non-normally distributed variables are reported as medians and interquartile ranges. Categorical variables are summarized as frequencies and percentages.

For group comparisons, independent samples t-tests were used for normally distributed continuous variables. The Mann–Whitney U test was used when normality assumptions were not met. The Kruskal–Wallis test was applied for comparisons involving more than two groups. Categorical variables were compared using the χ^2^ test or Fisher’s exact test, as appropriate.

To evaluate whether the associations between sex and liver biomarkers were independent of potential confounders, multivariable linear regression analyses were performed for GGT, AST/ALT ratio, and FIB-4. Models were adjusted for smoking status, educational level, alcohol consumption (weekly intake and maximum alcohol intake per occasion), and comorbidity burden. GGT and FIB-4 were log-transformed before analysis because of their skewed distributions. 

All tests were two-sided, and a *p*-value < 0.05 was considered statistically significant. Analyses were performed using Stata (v18) and RStudio (v4.4.0). 

## 3. Results

### 3.1. Characteristics of the Total Sample

The total sample included 931 participants, of whom 513 were women (55%), with a mean age of 71 ± 5 years. Overall, 63% of the participants consumed alcohol, with 78% of these individuals exhibiting risky consumption patterns; among participants with risky alcohol consumption, 59% were men. 

The median number of medications consumed per participant was 2, with antihypertensive medications being the most prevalent (51%), followed by analgesics (32%) and anxiolytics, antiepileptics, and hypnotics (23%). The number of medications was significantly lower among participants with non-risky alcohol consumption (*p* < 0.01). Individuals who did not consume alcohol had a higher prevalence of polypharmacy (5% vs. 2–3%; *p* < 0.01). The main descriptive characteristics are shown in Table 1.

### 3.2. Characteristics of Participants with Risky Alcohol Consumption

Table 2 shows the characteristics of participants with risky alcohol consumption (*n* = 455) according to sex. The female population constituted 41% of the sample. A statistically significant difference was identified in the mean SDU for both weekly and per-occasion consumption, with higher values observed in the male population (*p* < 0.01). Also, the proportion of current and former smokers was notably higher in the male group (*p* < 0.01).

Regarding the presence of comorbidities, the most prevalent were dyslipidemia (65%), hypertension (63%), and diabetes mellitus (25%). The mean total number of comorbidities was marginally higher among males (2.51 ± 1.43 vs. 2.14 ± 1.27, *p* = 0.02). Significant differences were observed in the prevalence by sex of heart disease (22% vs. 8%, *p* < 0.01), cancer (13% vs. 6%, *p* = 0.01), and chronic obstructive pulmonary disease (12% vs. 4%, *p* < 0.01).

**Figure 1:**
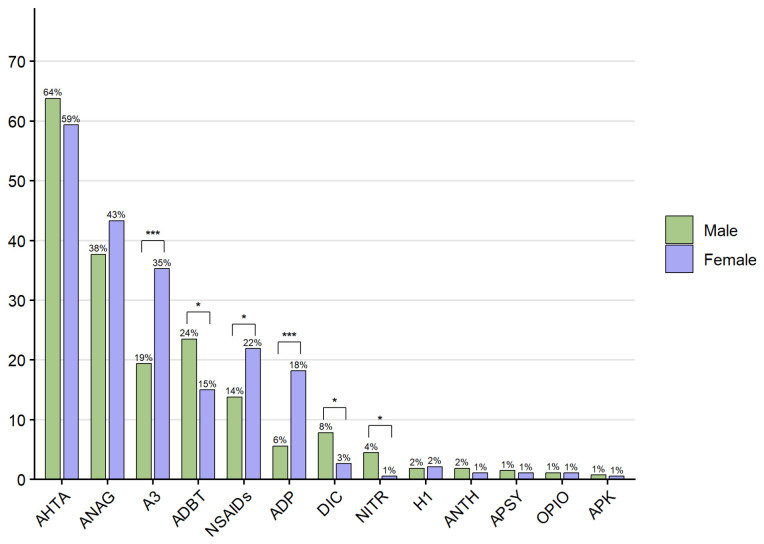
Medication use according to sex among participants with risky alcohol consumption (*n* = 455). Note: Medications not used by any participants (methotrexate and lithium carbonate) were excluded from the graph. * *p* < 0.05, *** *p* < 0.001. AHTA, Antihypertensive and alpha blockers; ANAG, Analgesics; A3, Anxiolytics, antiepileptics and hypnotics; ADBT, Antidiabetics; NSAIDs, Non-steroidal anti-inflammatory drugs; ADP, Antidepressants; DIC, Dicoumarinics; NITR, Nitrates; H1, Antihistaminics H1 and antiemetics; ANTH, Antihistamines antiallergics; APSY, Antipsychotics; OPIO, Morphine and opioids; APK, Antiparkinsonian.

Among participants with risky alcohol consumption, 10% were not taking any medication, 61% were taking one or two medications. A total of 62% of participants with risky alcohol consumption were prescribed antihypertensive medications or alpha blockers, followed by analgesics (40%), with no statistically significant differences observed based on sex. The analysis revealed that women had a similar mean number of drugs consumed (2.02 ± 1.15 vs. 1.83 ± 1.27, *p* = 0.08), with a significant increase in the use of anxiolytics, antiepileptics and hypnotics (35% vs. 19%, *p* < 0.01), anti-inflammatories (22% vs. 14%, *p* = 0.02), and antidepressants (18% vs. 6%, *p* < 0.01). Conversely, a higher proportion of male participants had increased consumption of antidiabetics (24% vs. 15%, *p* = 0.03), anticoagulants (8% vs. 3%, *p* = 0.02), and nitrates (4% vs. 1%, *p* = 0.01) (Figure 1 and Supplementary Table S2).

### 3.3. Blood Biochemistry Parameters of Participants with Risky Alcohol Consumption

**Figure 2:**
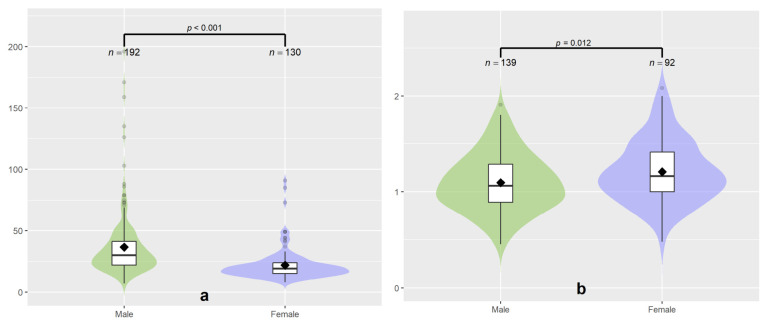
(**a**) Distribution of Gamma-glutamyl transferase (U/L) by sex (*n* = 322; male = 192, female = 130). (**b**) AST/ALT ratio or De Ritis index by sex (*n* = 231; male = 139, female = 92).

Mean GGT levels were significantly higher in men than in women: 41.52 ± 44.31 U/L vs. 21.88 ± 12.57 U/L, respectively (*p* < 0.001) (Figure 2a). This association remained significant after adjustment for smoking status, educational level, alcohol consumption, and comorbidity burden (β = −0.34, *p* < 0.01; Supplementary Table S3).

The AST/ALT ratio (De Ritis index) was also significantly higher in women than in men (1.24 vs. 1.10, *p* = 0.01) (Figure 2b). However, this association was no longer statistically significant after multivariable adjustment (Supplementary Table S3).

**Figure 3:**
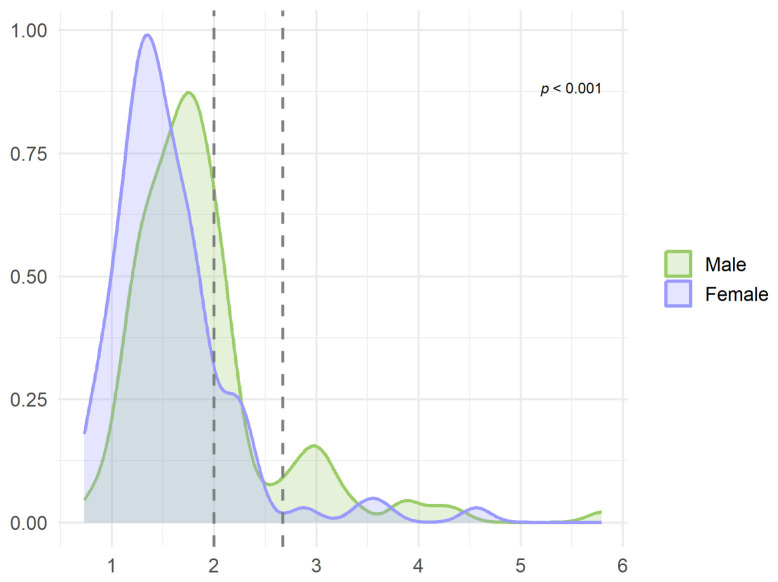
Distribution of FIB-4 by sex (*n* = 223; male = 131, female = 92). The dashed vertical lines indicate the age-adjusted FIB-4 cut-off values used in adults aged ≥ 65 years. An FIB-4 value < 2.0 rules out advanced liver fibrosis, whereas a value > 2.67 indicates a high risk of advanced liver fibrosis. Note: Participants with known chronic liver disease, viral or non-viral, were excluded from this analysis (*n* = 19).

Twenty-four percent of the participants had FIB-4 scores above 2.0, while 10% had scores above 2.67. After excluding participants with chronic liver disease, these prevalences decreased slightly to 22% and 9%, respectively. A statistically significant difference was identified between male and female subjects (*p* < 0.001). Specifically, 13% of male subjects demonstrated scores above 2.67, while this proportion was 4% among female subjects (Figure 3). Similarly, female sex remained independently associated with lower FIB-4 values after adjustment for potential confounders (β = −0.21, *p* < 0.01; Supplementary Table S3). 

## 4. Discussion

Our findings indicate a significant proportion of older adults with risky alcohol consumption who are simultaneously receiving pharmacological treatment. This coexistence is clinically relevant, as it may increase the potential for alcohol–drug interactions and susceptibility to adverse effects described in the literature [36]. Lower levels of alcohol consumption have been associated with a higher frequency of adverse drug reactions, with some evidence indicating that this risk could be greater in women; however, sex-specific data remain limited [18,19].

Consistent with previous studies, men reported higher alcohol consumption than women, both weekly and per occasion [5,37]. However, recent evidence suggests that alcohol consumption among women has increased over time [38,39,40,41]. Despite reporting lower levels of consumption, women may be more susceptible to the effects of alcohol due to physiological and metabolic differences. Specifically, women exhibit lower levels of gastric alcohol dehydrogenase activity, a lower proportion of total body water, and a higher percentage of adipose tissue, which contributes to higher blood alcohol concentrations [42,43,44]. In addition, women appear to be more biologically vulnerable to alcohol-related liver damage than men at comparable levels of alcohol exposure, potentially placing them at increased risk even with moderate alcohol consumption in older age [45,46,47]. 

Approximately 90% of participants were taking at least one medication, with antihypertensive drugs and analgesics being the most frequently prescribed. Moreover, almost 60% of the sample were taking two or more medications, and 2.9% fulfilled criteria for polypharmacy (≥5 drugs), highlighting a subgroup that may be particularly vulnerable to potential alcohol–drug interactions and adverse events reported in previous studies [48,49].

Although men had a higher burden of comorbidities, women tended to use a higher number of medications, although this difference did not reach statistical significance. Distinct medication use patterns were nevertheless observed according to sex. Women more frequently used medications related to pain management and mood disorders, whereas men were more likely to receive treatments aimed at the management and prevention of cardiovascular diseases. These findings are consistent with the distribution of comorbidities observed in our sample and with previous epidemiological evidence [49,50]. The trend towards a higher pharmacological burden among women, despite a lower comorbidity burden, has also been described previously and may be partially explained by sex differences in healthcare use, symptom reporting, and the higher prevalence of affective and somatic conditions among women [51,52]. However, because these comparisons were based on unadjusted analyses, they should be interpreted with caution, as residual confounding cannot be excluded. Therefore, the extent to which the observed differences are independently attributable to sex cannot be determined.

These differences in disease profiles and prescribing patterns underscore the importance of considering potential sex-specific consequences of alcohol use in older adults receiving pharmacological treatment. Previous evidence has shown that the concomitant use of alcohol and anxiolytics, hypnotics, antidepressants, and other central nervous system (CNS) depressants may increase the risk of sedation, cognitive impairment, and falls [36,53,54,55,56]. Likewise, the combined use of NSAIDs and alcohol has been associated with an elevated risk of gastric mucosal injury and gastrointestinal bleeding [56,57,58]. In men, who more frequently used cardiovascular medications, alcohol consumption in combination with these treatments has been related to adverse outcomes such as hypoglycemia in patients receiving antidiabetic treatment [23,48,56,59], potentiation of the blood pressure-lowering effects of antihypertensive medications [13,59,60,61], and increased bleeding risk in individuals receiving low-dose aspirin [33,56,59,62]. Overall, our findings suggest that older women with risky alcohol consumption may represent a subgroup potentially more vulnerable to alcohol–medication interactions involving CNS-active or anti-inflammatory drugs, based on their medication use profile. In contrast, older men may present a medication profile theoretically associated with a higher risk of alcohol-related complications involving cardiovascular pharmacotherapy. This interpretation is consistent with previous studies identifying cardiovascular and CNS medications as the drug classes most frequently involved in potentially serious alcohol–medication interactions among older adults [63,64]. However, because clinically relevant alcohol–medication interactions and their associated adverse outcomes were not directly assessed, these findings should not be interpreted as evidence that such interactions occurred in our study population. 

When we analyzed serological markers associated with alcohol consumption, mean GGT levels remained below clinically relevant thresholds in both sexes. Nevertheless, men showed higher GGT levels than woman, consistent with previous studies reporting a stronger association between alcohol intake and GGT elevation in males, although GGT is a non-specific biomarker that may also be elevated in several non-alcohol-related conditions [65,66]. Nevertheless, previous studies have shown that women may exhibit elevated GGT levels even at lower levels of alcohol consumption, suggesting a greater biological susceptibility to alcohol-related liver injury [65,67,68]. The AST/ALT ratio was greater than 1 in both sexes and significantly higher in women in the unadjusted analysis, although values remained below those commonly associated with advanced alcohol-related liver disease [69,70]. However, this association was no longer statistically significant after adjustment for smoking status, educational level, alcohol consumption, and comorbidity burden. Moreover, because both AST and ALT values remained within normal ranges, the small difference observed in the AST/ALT ratio is unlikely to be clinically meaningful. Therefore, these findings should be interpreted cautiously and warrant further investigation in longitudinal studies assessing clinically relevant liver outcomes.

Finally, alcohol consumption is a well-established risk factor for the development of fibrosis, cirrhosis and hepatocellular carcinoma (HCC) [33]. Biological and metabolic differences appear to make women more susceptible to alcohol-induced liver damage and, therefore, more likely to develop HCC than men [46,47,71]. In contrast, we observed a higher prevalence of elevated FIB-4 values among men. Similar sex-related differences in FIB-4 values have also been reported in individuals without known liver disease [72]. This apparent inconsistency may reflect the fact that FIB-4 is an indirect surrogate marker of liver fibrosis based on age, AST, ALT, and platelet count, rather than a direct measure of biological susceptibility to alcohol-induced liver injury. To our knowledge, there are currently no precise data regarding the prevalence of liver fibrosis among adults aged >65 years with risky alcohol consumption. Previous studies have reported that approximately 20% of adults with risky alcohol consumption, without age restrictions, present markers suggestive of advanced fibrosis [33,73]. In our cohort, 24% of participants had FIB-4 values above 2.0 and 10% above 2.67. These proportions decreased only slightly after excluding participants with previously diagnosed chronic liver disease, suggesting that most individuals with elevated FIB-4 values had no previous diagnosis of liver disease. Although FIB-4 is a useful non-invasive tool for ruling out advanced fibrosis [35], its accuracy may be limited by age, comorbidities, and the use of certain medications [72,74]. Therefore, although elevated FIB-4 values may identify individuals who warrant further evaluation, they should be interpreted as a screening signal rather than evidence of advanced liver fibrosis and should ideally be confirmed using more specific diagnostic methods. 

This study has several limitations that should be considered when interpreting the findings. Firstly, due to its cross-sectional design, no temporal or causal inferences can be made between risky alcohol consumption, medication use patterns, comorbidities, and hepatic biomarkers. Therefore, the observed sex-related differences should be interpreted as associations rather than evidence of causality. Secondly, the definition of risky alcohol consumption used in the ALANE cohort may have increased the heterogeneity within the study population because it combined quantitative alcohol consumption thresholds with clinical criteria related to alcohol-sensitive medical conditions and alcohol-interacting medications. Consequently, the findings should be interpreted within the context of this definition and may not be directly generalizable to studies using alcohol consumption thresholds alone. Thirdly, as the study was based on data from a cohort recruited more than a decade ago, alcohol consumption behaviors and prescribing patterns may have changed over time. Nevertheless, this is a well-characterized community-based sample, and our findings are consistent with the available literature. Fourthly, the study was not originally designed to characterize medication use, comorbidities, or hepatic biomarkers, limiting our ability to explore associations in greater depth and to directly assess clinically relevant alcohol–drug interactions and related adverse outcomes. Furthermore, the lack of biochemical data from participants with lower-risk alcohol consumption or abstainers precluded direct comparisons across alcohol consumption groups. Finally, hepatic fibrosis was assessed using non-invasive serological indices calculated retrospectively from previously collected data, without confirmation by a reference standard. Although age-adjusted cut-offs were applied, the diagnostic performance of FIB-4 may still be influenced by advanced age. Despite these limitations, this study provides relevant insight into sex-related differences in medication use, comorbidities, and hepatic biomarkers among older adults with risky alcohol consumption, supporting future sex-specific research in this population.

## 5. Conclusions

This study identified important sex-related differences in medication use, comorbidities, and hepatic biomarkers among older adults with risky alcohol consumption. These findings highlight the importance of routinely assessing alcohol consumption and reviewing medication use in primary care to identify individuals at increased risk of alcohol–medication interactions and alcohol-related liver disease. Incorporating a sex-specific perspective into both clinical practice and future longitudinal research may improve the identification, prevention, and management of older adults at greater risk of alcohol-related adverse health outcomes.

## Figures and Tables

**Table 1:** Characteristics of the total sample (*n* = 931).

	Abstainer (*n* = 349)	Non-Risky Alcohol Consumption (*n* = 127)	Risky Alcohol Consumption (*n* = 455)	Total (*n* = 931)	*p*
**Sex**					
Male	88 (25.2%)	62 (48.8%)	268 (58.9%)	418 (44.9%)	<0.01
Female	261 (74.8%)	65 (51.2%)	187 (41.1%)	513 (55.1%)	
**Age**	70.0 (67.5–75.1)	70.0 (67.8–75.4)	69.8 (67.8–72.8)	70.0 (67.7–74.1)	0.30
**Educational level**					
No formal education	127 (36.6%)	33 (26.8%)	88 (19.8%)	248 (27.1%)	<0.01
Primary education	172 (49.6%)	71 (57.7%)	238 (53.6%)	481 (52.6%)	
Secondary or higher education	48 (13.8%)	19 (15.5%)	118 (26.6%)	185 (20.2%)	
**Alcohol consumption**					
SDU per week	0 (0)	1 (0–3)	7 (2–14)	0 (0–7)	<0.01
SDU per time	0 (0)	1 (1–1)	2 (1–2)	1 (0–2)	<0.01
**Number of drugs**					
0	114 (32.7%)	92 (72.4%)	45 (9.9%)	251 (27.0%)	<0.01
1	51 (14.6%)	17 (13.4%)	146 (32.1%)	214 (23.0%)	
2	74 (21.2%)	8 (6.3%)	133 (29.2%)	215 (23.1%)	
3	55 (15.8%)	6 (4.7%)	83 (18.2%)	144 (15.5%)	
4	36 (10.3%)	2 (1.6%)	35 (7.7%)	73 (7.8%)	
≥5	19 (5.44%)	2 (1.6%)	13 (2.9%)	34 (3.7%)	
**Drugs consumption**					
NSAIDs	62 (17.8%)	16 (12.6%)	78 (17.1%)	156 (16.8%)	0.39
Analgesics	107 (30.7%)	8 (6.3%)	182 (40.0%)	297 (31.9%)	<0.01
Nitrates	12 (3.4%)	1 (0.8%)	13 (2.9%)	26 (2.8%)	0.33
Dicoumarinics	19 (5.4%)	1 (0.8%)	26 (5.7%)	46 (4.9%)	0.04
Antidepressants	49 (14.0%)	2 (1.6%)	49 (10.8%)	100 (10.7%)	<0.01
A3	89 (25.5%)	5 (3.9%)	118 (25.9%)	212 (22.8%)	<0.01
ANTH	3 (0.9%)	2 (1.6%)	7 (1.5%)	12 (1.3%)	0.66
Antipsychotics	6 (1.7%)	0 (0%)	6 (1.3%)	12 (1.3%)	0.44
Antiparkinsonian	0 (0%)	0 (0%)	3 (0.7%)	3 (0.3%)	0.38
H1	8 (2.3%)	2 (1.6%)	9 (2.0%)	19 (2.0%)	0.95
Opioids	8 (2.3%)	4 (3.2%)	5 (1.1%)	17 (1.8%)	0.20
Methotrexate	1 (0.3%)	1 (0.8%)	0 (0%)	2 (0.2%)	0.12
Antidiabetics	72 (20.6%)	8 (6.3%)	91 (20.0%)	171 (18.4%)	<0.01
HTA	173 (49.6%)	19 (15.0%)	282 (62.0%)	474 (50.9%)	<0.01

Note: NSAIDs, Non-steroidal anti-inflammatory drugs; A3, Anxiolytics, antiepileptics and hypnotics; ANTH, Antihistamines antiallergics; H1, Antihistaminics H1 and antiemetics; HTA, Antihypertensive and alpha blockers. Categorical variables are presented as n (%), and continuous variables as mean ± standard deviation (normally distributed variables) or median (interquartile range) (non-normally distributed variables).

**Table 2:** Characteristics of participants with risky alcohol consumption by sex (*n* = 455).

	Female (*n* = 187)	Male (*n* = 268)	Total (*n* = 455)	*p*
**Age**	69.59 (67.59–72.12)	70.25 (67.83–73.18)	69.84 (67.78–72.80)	0.14
**Educational level**				
No formal education	45 (24.7%)	43 (16.4%)	88 (19.8%)	0.05
Primary education	97 (53.3%)	141 (53.8%)	238 (53.6%)	
Secondary or higher education	40 (22.0%)	78 (29.8%)	118 (26.6%)	
**BMI**	29.2 ± 4.5	29.0 ± 3.9	29.1 ± 4.2	0.72
**Tabaco**				
No smoker	154 (88.0%)	56 (22.6%)	210 (49.7%)	<0.01
Ex-smoker	14 (8.0%)	145 (58.5%)	159 (37.6%)	
Smoker	7 (4.0%)	47 (19.0%)	54 (12.8%)	
**Alcohol consumption**				
SDU per week	2 (1–7)	9 (6–16)	7 (2–14)	<0.01
SDU per time	1 (1–2)	2 (1–3)	2 (1–2)	<0.01
**Number of drugs**				
0	8 (4.3%)	37 (13.8%)	45 (9.9%)	
1	65 (34.8%)	81 (30.2%)	146 (32.1%)	
2	56 (30.0%)	77 (28.7%)	133 (29.3%)	0.08
3	37 (19.8%)	46 (17.2%)	83 (18.2%)	
4	15 (8.0%)	20 (7.5%)	35 (7.7%)	
≥5	6 (3.21%)	7 (2.6%)	13 (2.9%)	
**Number of comorbidities**	2 (1–3)	2 (2–3)	2 (1–3)	0.02
**Comorbidities**				
Hypertension	107 (60.1%)	161 (64.4%)	268 (62.6%)	0.37
Diabetes Mellitus	40 (22.5%)	69 (27.6%)	109 (25.5%)	0.23
Dyslipidemia	122 (68.5%)	158 (63.2%)	289 (65.4%)	0.25
Obesity	41 (23.0%)	61 (24.4%)	102 (23.8%)	0.74
Heart disease	15 (8.4%)	54 (21.6%)	69 (16.1%)	<0.01
Cerebrovascular accident	6 (3.4%)	15 (6.0%)	21 (4.9%)	0.26
Psychiatric disorders	16 (9.0%)	11 (4.4%)	27 (6.3%)	0.05
Peripheral artery disease	1 (0.6%)	9 (3.6%)	10 (2.3%)	0.05
Non-viral CLD	3 (1.7%)	10 (4.0%)	13 (3.0%)	0.25
CLD	2 (1.1%)	5 (2.0%)	7 (1.6%)	0.70
Gastroduodenal ulcer	3 (1.7%)	7 (2.8%)	10 (2.3%)	0.53
Bronchial asthma	8 (4.5%)	4 (1.6%)	12 (2.8%)	0.08
COPD	7 (3.9%)	31 (12.4%)	38 (8.9%)	<0.01
Cancer	10 (5.6%)	32 (12.8%)	42 (9.8%)	0.01

Note: BMI, Body Mass Index; CLD, Chronic Liver Disease; COPD, Chronic Obstructive Pulmonary Disease. Categorical variables are presented as n (%), and continuous variables as mean ± standard deviation (normally distributed variables) or median (interquartile range) (non-normally distributed variables).

## Data Availability

The data presented in this study are available on request from the corresponding author due to ethical and privacy restrictions.

## Supplementary Materials

The following supporting information can be downloaded at: https://www.mdpi.com/article/10.3390/nu18142346/s1, Table S1: Classification of participants according to the criteria used to define risky alcohol consumption in the ALANE cohort; Table S2: Medication use according to sex among participants with risky alcohol consumption (*n* = 455); Table S3: Models adjusted for smoking status, educational level, alcohol consumption and comorbidity burden.

## Abbreviations

The following abbreviations are used in this manuscript:

SDU	Standard Drink Units
NSAIDs	Nonsteroidal anti-inflammatory drugs
PHCs	Primary Health Care centers
ICD-10	International Classification of Disease
AST	Aspartate aminotransferase
ALT	Alanine aminotransferase
GGT	Gamma-glutamyl transferase
SIAP	Primary Care Information System
A3	Anxiolytics, antiepileptics and hypnotics
ANTH	Antihistamines antiallergics
H1	Antihistaminics H1 and antiemetics
OPIO	Morphine and opioids
HTA	Antihypertensive and alpha blockers
BMI	Body Mass Index
CLD	Chronic Liver Disease
COPD	Chronic Obstructive Pulmonary Disease
ANAG	Analgesics
ADBT	Antidiabetics
ADP	Antidepressants
DIC	Dicoumarinics
NITR	Nitrates
APSY	Antipsychotics
APK	Antiparkinsonian
CNS	Central Nervous System
HCC	Hepatocellular Carcinoma
