# Supplementary material for: Sex Differences in Older Adults with Risky Alcohol Consumption: Medication Use, Comorbidities, and Hepatic Biomarkers
Source: Nutrients. 2026 Jul 17;18(14):2346. doi: 10.3390/nu18142346 (PMC13414805; doi:10.3390/nu18142346)
Supplement: Supplementary file 1 [file nutrients-18-02346-s001.zip › nutrients-4376738-supplementary.pdf]

Supplementary Materials

Table S1. Classification of participants according to the criteria used to define risky alcohol consumption in the ALANE cohort.

Risky drinking category	Definition	Female (*n* = 187)	Male (*n* = 268)	Total (*n* = 455)
Low alcohol consumption + medication/condition	Alcohol consumption below the quantitative risk thresholds in participants with alcohol-interacting medications and/or alcohol-sensitive medical conditions	87 (46.5%)	80 (29.9%)	167 (36.7%)
Occasional alcohol consumption + medication/condition	Occasional alcohol consumption below the quantitative risk thresholds in participants with alcohol-interacting medications and/or alcohol-sensitive medical conditions	36 (19.3%)	21 (7.8%)	57 (12.5%)
Heavy episodic drinking	Alcohol consumption exceeding the threshold for a single drinking occasion	2 (1.1%)	6 (2.2%)	8 (1.8%)
Heavy episodic drinking + medication/condition	Heavy episodic drinking in participants with alcohol-interacting medications and/or alcohol-sensitive medical conditions	38 (20.3%)	8 (3.0%)	46 (10.1%)
Chronic high-risk alcohol consumption	Average alcohol consumption exceeding the weekly and/or daily quantitative risk thresholds	6 (3.2%)	31 (11.6%)	37 (8.1%)
Chronic high-risk alcohol consumption + medication/condition	Chronic high-risk alcohol consumption in participants with alcohol-interacting medications and/or alcohol-sensitive medical conditions	18 (9.6%)	122 (45.5%)	140 (30.8%)

Table S2. Medication use according to sex among participants with risky alcohol consumption (*n* = 455).

	Female (*n* = 187)	Male (*n* = 268)	Total (*n* = 455)	*p*
AHTA	111 (59.4%)	171 (63.8%)	282 (62.0%)	0.34
ANAG	81 (43.3%)	101 (37.7%)	182 (40.0%)	0.23
A3	66 (35.3%)	52 (19.4%)	118 (25.9%)	< 0.01
ADBT	28 (15.0%)	63 (23.5%)	91 (20.0%)	0.03
NSAIDs	41 (21.9%)	37 (13.8%)	78 (17.1%)	0.02
ADP	34 (18.2%)	15 (5.6%)	49 (10.8%)	< 0.01
DIC	5 (2.7%)	21 (7.8%)	26 (5.7%)	0.02
NITR	1 (0.5%)	12 (4.5%)	13 (2.9%)	0.01
H1	4 (2.1%)	5 (1.9%)	9 (2.0%)	0.84
ANTH	2 (1.1%)	5 (1.9%)	7 (1.5%)	0.50
APSY	2 (1.1%)	4 (1.5%)	6 (1.3%)	0.70
OPIO	2 (1.1%)	3 (1.12%)	5 (1.1%)	0.96
APK	1 (0.5%)	2 (0.8%)	3 (0.7%)	0.78

Note: Medications not used by any participants (methotrexate and lithium carbonate). AHTA, Antihypertensive and alpha blockers; ANAG, Analgesics; A3, Anxiolytics, antiepileptics and hypnotics; ADBT, Antidiabetics; NSAIDs, Non-steroidal anti-inflammatory drugs; ADP, Antidepressants; DIC, Dicoumarinics; NITR, Nitrates; H1, Antihistaminics H1 and antiemetics; ANTH, Antihistamines antiallergics; APSY, Antipsychotics; OPIO, Morphine and opioids; APK, Antiparkinsonian.

Table S3. Models adjusted for smoking status, educational level, alcohol consumption and comorbidity burden.

Outcome	Adjusted β (Female vs Male)	95% CI	*p*
Log GGT	-0.34	-0.50 to 0.17	<0.01
AST/ALT ratio	0.08	-0.07 to 0.23	0.29
Log FIB-4	-0.21	-0.34 to -0.08	<0.01
